# The Ideal Car for General Practice

**Published:** 1912-01-13

**Authors:** 


### THE IDEAL CAR FOR GENERAL PRACTICE.

The cars sold at the present day for touring purposes are not suitable for the rough work consequent on being used for a general practice. The body work is too flimsy, the steel work is too finely cut down to give the maximum of result for the minimum of weight, that they are soon nothing less than "travelling rattles." In order to secure a car suitable for three or four years' hard work in all weathers, and over all conditions of roads, it is necessary to pay attention to the following points amongst others.

The chassis should be a very strong one, and fairly solidly made of good steel. The tyres should be as large in tread as can be fitted to the machine. The steering should be strong, irreversible, and provided with means to take up any wear that occurs, however trifling. The springing should be exceptionally strong, and a good tip is to insist on springs half as strong again as the manufacturer recommends being fitted, and the chassis should also be fitted with one of the best types of shock absorber obtainable.

The body work should be very strong, and also of the very best seasoned timber. Leather upholstery should be of the best quality. The colour scheme should be such that after a long day in the country or town on muddy roads the car looks fairly clean. Mudguards of extra width and special design aid this very considerably. The radiator should be placed directly in front of the dash-board, and specially long dumb-irons fitted in order to protect the more special parts of the car if it should collide with cattle, etc., during a night call.

The cooling system should be such that there is ample water-supply and a good enough pump to keep the car satisfactorily cool if run for three hours on end all out on the track with the car overloaded as regards its normal carrying weight.

### What Horse-Power?

The engine should be of sufficient power to negotiate all the hills within fifteen miles of the centre of the practice at twenty miles per hour in any weather. It can be of two, four, or six cylinders, according to choice, but it must be borne in mind that the expenses of upkeep and running vary directly as the square of the number of cylinders—thus, four cylinders are four times as costly to run as two, and six cylinders in the ratio of nine to four as compared with a four-cylinder car.

The engine requires also to be absolutely reliable, and should be capable of running for three months at its ordinary rate of working, without requiring any adjustments of any sort. There should be sliding or revolving sleeves, or a two-stroke engine, so as to get rid of two faults of a poppet-valve engine, viz., pitting of the valve and its seating, and breaking of the stem or head.

The clutch should be a multiple plate one, and should be kept scrupulously clean after being received, and cleaned out and oiled every three weeks. The gear-box should be easily accessible for cleaning and repairs. The gears should be of the finest possible steel, carefully cut, and specially case-hardened.

The transmission should be through differential, and then by chain drives, running in oil baths, to the wheels. This obviates the great danger of breakage in the live axle on very rough roads, such as a medical man travels daily, but which no self-respecting chauffeur would willingly tackle to save even such a distance as five miles by the main road.

### A Self-Starting Device.

The car requires to be fitted with a real dual ignition system, each working independently of the other, even to the extent of double series of sparking plugs. The car also requires to be fitted with some sort of self-starting device which can be actuated from the driving seat, and act whether the engine is hot, or has been standing cold for several hours.

The finished car should be as light as possible, at the same time as it conforms to the foregoing essential characteristics.

Commercial vehicles, such as lorries, delivery vans, motor 'buses, etc., have had much ingenuity and time expended on them to make them a paying success.

It is time that the manufacturers woke up to the fact that there is another type of special car required besides their touring models. Such a car would necessarily not be a cheap one, but the purchaser would be amply rewarded for his extra outlay by reliability, comfort, and lasting qualities, combined with a greatly diminished repair and running bill, allowing for the slightly increased cost of large tyres, and the extra petrol needed beyond what would be required by the same powered "touring" car.

We trust that before the next show there will be a fair number of these special cars for sale, as the makers would be well repaid for the necessary outlay.

